# Integrating bioinformatics and machine learning to identify AhR-related gene signatures for prognosis and tumor microenvironment modulation in melanoma

**DOI:** 10.3389/fimmu.2024.1519345

**Published:** 2025-01-06

**Authors:** Qianru Li, Heli Li

**Affiliations:** ^1^ Department of Dermatology, Wuhan No.1 Hospital, Wuhan, Hubei, China; ^2^ Hubei Province & Key Laboratory of Skin Infection and Immunity, Wuhan, Hubei, China; ^3^ Division of Child Healthcare, Department of Pediatrics, Tongji Hospital, Tongji Medical College, Huazhong University of Science and Technology, Wuhan, China

**Keywords:** Aryl Hydrocarbon Receptor, melanoma, prognostic model, immune cell infiltration, machine learning models

## Abstract

**Background:**

The Aryl Hydrocarbon Receptor (AhR) pathway significantly influences immune cell regulation, impacting the effectiveness of immunotherapy and patient outcomes in melanoma. However, the specific downstream targets and mechanisms by which AhR influences melanoma remain insufficiently understood.

**Methods:**

Melanoma samples from The Cancer Genome Atlas (TCGA) and normal skin tissues from the Genotype-Tissue Expression (GTEx) database were analyzed to identify differentially expressed genes, which were intersected with a curated list of AhR-related pathway genes. Prognostic models were subsequently developed, and feature genes were identified. Advanced methodologies, including Gene Set Enrichment Analysis (GSEA) and immune cell infiltration analysis, were employed to explore the biological significance of these genes. The stability of the machine learning models and the relationship between gene expression and immune infiltrating cells were validated using three independent melanoma datasets. A mouse melanoma model was used to validate the dynamic changes of the feature genes during tumor progression. The relationship between the selected genes and drug sensitivity, as well as non-coding RNA interactions, was thoroughly investigated.

**Results:**

Our analysis identified a robust prognostic model, with four AhR-related genes (MAP2K1, PRKACB, KLF5, and PIK3R2) emerging as key contributors to melanoma progression. GSEA revealed that these genes are involved in primary immunodeficiency. Immune cell infiltration analysis demonstrated enrichment of CD4^+^ naïve and memory T cells, macrophages (M0 and M2), and CD8^+^ T cells in melanoma, all of which were associated with the expression of the four feature genes. Importantly, the diagnostic power of the prognostic model and the relevance of the feature genes were validated in three additional independent melanoma datasets. In the mouse melanoma model, Map2k1 and Prkacb mRNA levels exhibited a progressive increase with tumor progression, supporting their role in melanoma advancement.

**Conclusions:**

This study presents a comprehensive analysis of AhR-related genes in melanoma, highlighting MAP2K1, PRKACB, KLF5, and PIK3R2 as key prognostic markers and potential therapeutic targets. The integration of bioinformatics and machine learning provides a robust framework for enhancing prognostic evaluation in melanoma patients and offers new avenues for the development of treatments, particularly for those resistant to current immunotherapies.

## 1 Introduction

Melanoma, a highly aggressive skin cancer type, accounts for around 4% of skin tumor cases but is responsible for approximately 80% of skin cancer-related deaths (1). Immunotherapy, especially through immune checkpoint inhibitors, has transformed the treatment landscape for melanoma, providing substantial benefits to certain patient groups (2). However, many patients exhibit variable responses or develop resistance to these therapies, highlighting the need for more precise therapeutic strategies. Recent oncology research has increasingly focused on the aryl hydrocarbon receptor (AhR), historically known for its role in xenobiotic metabolism (3). Environmental carcinogens, such as polycyclic aromatic hydrocarbons and polychlorinated biphenyls, act as ligands that bind to and activate AhR (4). In melanoma, AhR is implicated in multiple chemical carcinogenic signaling pathways and exhibits a dual role, functioning as both a promoter and suppressor of tumorigenesis (5). It has also emerged as a key modulator within the tumor microenvironment (TME) (6).

Studies suggest that AhR activation triggers the expression of various cytokines and immune-modulating factors, shaping the TME in distinct ways (7). In melanoma, AhR activation has been linked to the recruitment and activity of regulatory T cells (Tregs), which suppress anti-tumor immunity (8). Conversely, AhR signaling has also been shown to enhance anti-tumor immune responses by promoting Th17 cell differentiation (9), highlighting its complex role. Additionally, AhR activation can drive macrophages to acquire an immunosuppressive phenotype, which can mediate chemotherapy resistance in tumor (10, 11). AhR is notably present in various crucial immune cells, both innate and adaptive immunity, but comprehensive analyses of the relevant pathway are lacking (4). This gap in knowledge presents a significant hurdle in harnessing AhR’s potential in therapeutic strategies.

While several studies have associated AhR with melanoma prognosis and resistance to immune checkpoint inhibitors (12), significant gaps persist. For instance, although AHR is implicated in the recruitment of immunosuppressive cells (3), the key downstream targets of the AhR pathway in melanoma, their roles in shaping the tumor microenvironment, and their potential as reliable prognostic markers have yet to be fully characterized. This study addresses these gaps by leveraging bioinformatics tools to systematically analyze AhR-related genes and their associations with melanoma. Through robust machine learning models, it identifies novel prognostic markers and potential therapeutic targets. By enhancing our understanding of the AhR pathway, this research provides a foundation for identifying new treatment strategies and clarifying the biological mechanisms driving melanoma progression.

## 2 Materials and methods

### 2.1 Raw data acquisition

**Figure 1:**
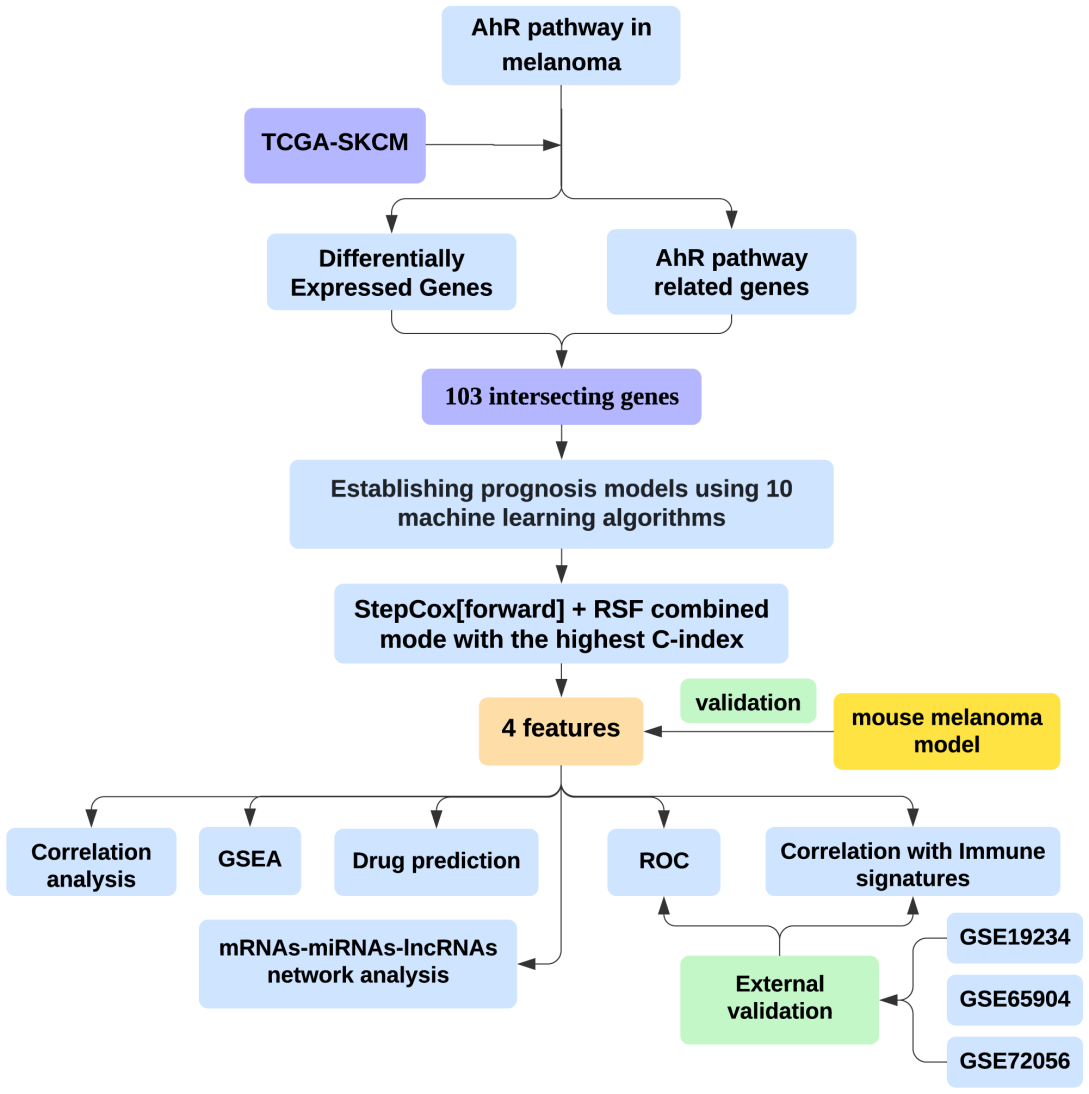
Integrating bioinformatics and machine learning to identify AhR-related gene signatures for prognosis and tumor microenvironment modulation in melanoma.

**Figure 1** was created to show the flowchart of our data analysis process. The study utilized public datasets from TCGA (www.cancer.gov) for melanoma samples and GTEx (www.gtexportal.org) as controls. The combined data enabled the comparison of melanoma-specific gene expression patterns. Gene expression and single-cell RNA sequencing (scRNA-seq) data for melanoma were obtained from the Gene Expression Omnibus (www.ncbi.nlm.nih.gov/geo/). The GSE19234 dataset (GPL570) includes data from 38 melanoma patients (13). The GSE65904 dataset (GPL10558) consists of data from 214 melanoma patients, with only cutaneous melanoma samples that have available disease-specific survival information and survival duration selected (n=21) (14). The GSE72056 dataset (GPL18573) contains single-cell RNA-seq data from 4645 cells isolated from 19 melanoma patients (15). Gene expression across different cell types was processed and analyzed using the Seurat and SingleR R packages (16, 17).

### 2.2 Differentially expressed genes

Using the limma package (18), DEGs were screened according to the threshold parameters (|logFC| > 0.5 and adjusted p < 0.05). Volcano plots and heatmaps were generated to show the gene expression changes by R. The analysis focused on genes within the chemical carcinogenesis receptor activation signaling pathway (hsa05207) involving AhR pathway, identifying 103 intersecting genes. Network of 103 intersecting genes were conducted by igraph package with p < 0.5, r > 0.3. The skin-specific coexpression network were conducted by NetworkAnalyst, which have human tissue-specific gene coexpression from the iNetModels database (19).

### 2.3 Construction of prognostic model and immune cell infiltration analysis in melanoma

The melanoma dataset from TCGA was split into training (75%) and testing (25%) sets. Using the R software and the Mime package, we constructed an optimal prognostic model and identified feature genes (20). Mime, a machine learning framework, integrates ten classic algorithms: Lasso, Enet, Boruta, CoxBoost, Random Forests (RSF), eXtreme Gradient Boosting (Xgboost), StepCox, plsRcox, Generalized Boosted Regression Model (GBM), and Support Vector Machine Recursive Feature Elimination (SVM-REF). A total of 117 combinations were applied with K-fold cross-validation for model training. The model’s performance, assessed using the C-index, demonstrated its capability to stratify patients into high- and low-risk survival groups. Immune cell infiltration was analyzed using xCell, Epic, abis, estimate, and Cibersort via the Mime and immunedeconv packages (21).

### 2.4 Gene set enrichment and variation analyses

GSEA was used to investigate the functional enrichment of the feature genes. We divided each feature gene into high expression and low expression groups based on its expression level, and conducted differential analysis with a p-valuecutoff value of 0.05. Using R software along with the enrichplot packages, KEGG pathways were identified to explore the roles of feature genes in TCGA melanoma samples (22).

### 2.5 Ear injection model in mice

C57BL/6 mice were purchased from HFK Bioscience (Beijing, China) and housed in a specific pathogen-free environment. For the ear injection model, B16-F10 cells (2 × 10^5^) were injected intradermally into the ears of mice (8-week-old) in 25 μl of HBSS buffer.

### 2.6 RNA extraction and quantitative real-time PCR

Total RNA from tumor or normal tissue was obtained. qRT-PCR mixtures were prepared by a SYBR green real-time PCR kit (Toyobo, Osaka, Japan). mRNA levels were normalized to GAPDH, and fold changes were determined using the 2^−ΔΔCt^ method. Primer pairs: 5’- TCTCCACACCTATGGTGCAA -3’ and 5’- CAAGAAACAGGGGAGCTGAG -3’ (Gapdh); 5’- AACGGTGGAGTGGTCTTCAAG -3’ and 5’- CGGATTGCGGGTTTGATCTC -3’ (Map2k1); 5’- AGGGCAGGACATGGACATTG -3’ and 5’- CGCCTTATTGTAACCCTTGCTG -3’ (Prkacb); 5’- CAGGCCACCTACTTTCCCC -3’ and 5’- GAATCGCCAGTTTGGAAGCAA -3’ (Klf5); 5’- ACCTAAGCCCTCTAAGGCAAA -3’ and 5’- TCCCGGAGTCTCTCATTCACC-3’ (Pik3r2).

### 2.7 Drug sensitivity

To explore the therapeutic implications, drug-gene interactions were predicted for the identified hub genes using Gene Set Cancer Analysis (GSCA). GSCA integrates over 750 small molecule drugs from Cancer Therapeutics Response Portal (CTRP) and Genomics of Drug Sensitivity in Cancer (GDSC) databases (23). This analysis is crucial for identifying potential compounds that could reverse resistance to immunotherapy.

### 2.8 mRNA-miRNA-lncRNA network

A comprehensive mRNA-miRNA-lncRNA network was constructed to explore the post-transcriptional regulation of the feature genes. Using the miRDB database (24) for miRNA prediction (target score ≥ 95) and ENCORI database (25) for lncRNA prediction.

## 3 Results

### 3.1 DEGs identification and correlation analysis

**Figure 2:**
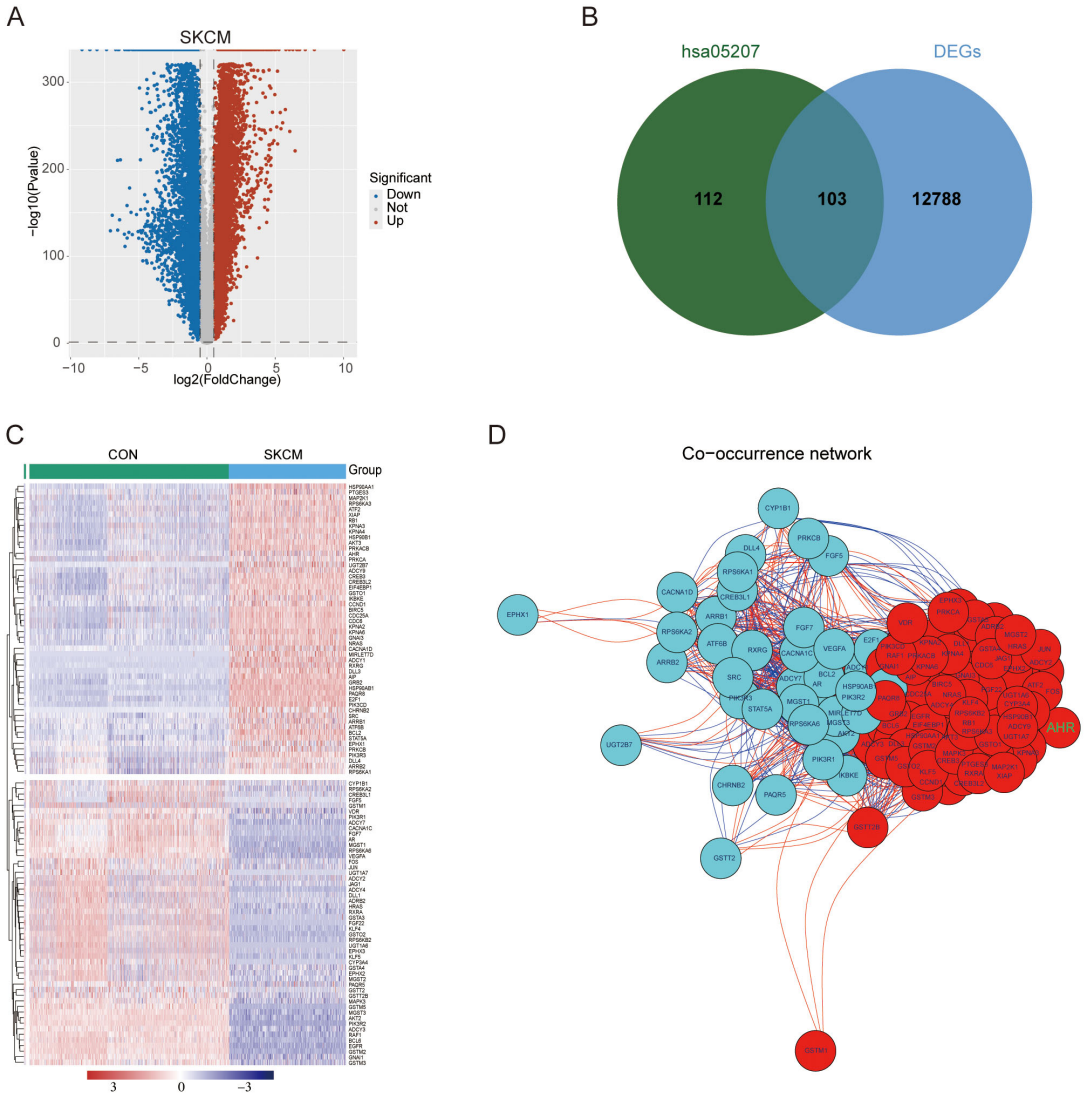
DEGs identification and correlation analysis. **(A)** Volcano plot of DEGs between melanoma samples from TCGA-SKCM and control tissues from GTEx. **(B)** 103 intersecting genes of DEGs and genes from the hsa05207 pathway. **(C)** Heat map depicting the expression patterns of DEGs across the two groups. **(D)** Network of 103 intersecting genes. Red nodes represent upregulated genes and blue nodes representing downregulated genes. Red lines indicate positive correlations, while blue lines denote negative correlations between the genes.

We conducted a differential gene expression analysis between melanoma samples from TCGA and normal control tissues from GTEx, identifying 12891 differentially expressed genes (DEGs) (**Figure 2A**). From the human chemical carcinogenesis receptor activation signaling pathway (hsa05207), which is closely associated with the AHR pathway, we identified 215 related genes (**Supplementary Figure S1**). Of these, 103 genes overlapped with the DEGs (**Figure 2B**), with 52 genes being upregulated and 51 downregulated in melanoma (**Figure 2C**). Correlation analysis further demonstrated strong interrelationships among these 103 genes (**Figure 2D**). Additionally, we constructed the skin-specific coexpression network of these 103 genes using NetworkAnalyst (**Supplementary Figure S2**).

### 3.2 Construction of prognostic models

**Figure 3:**
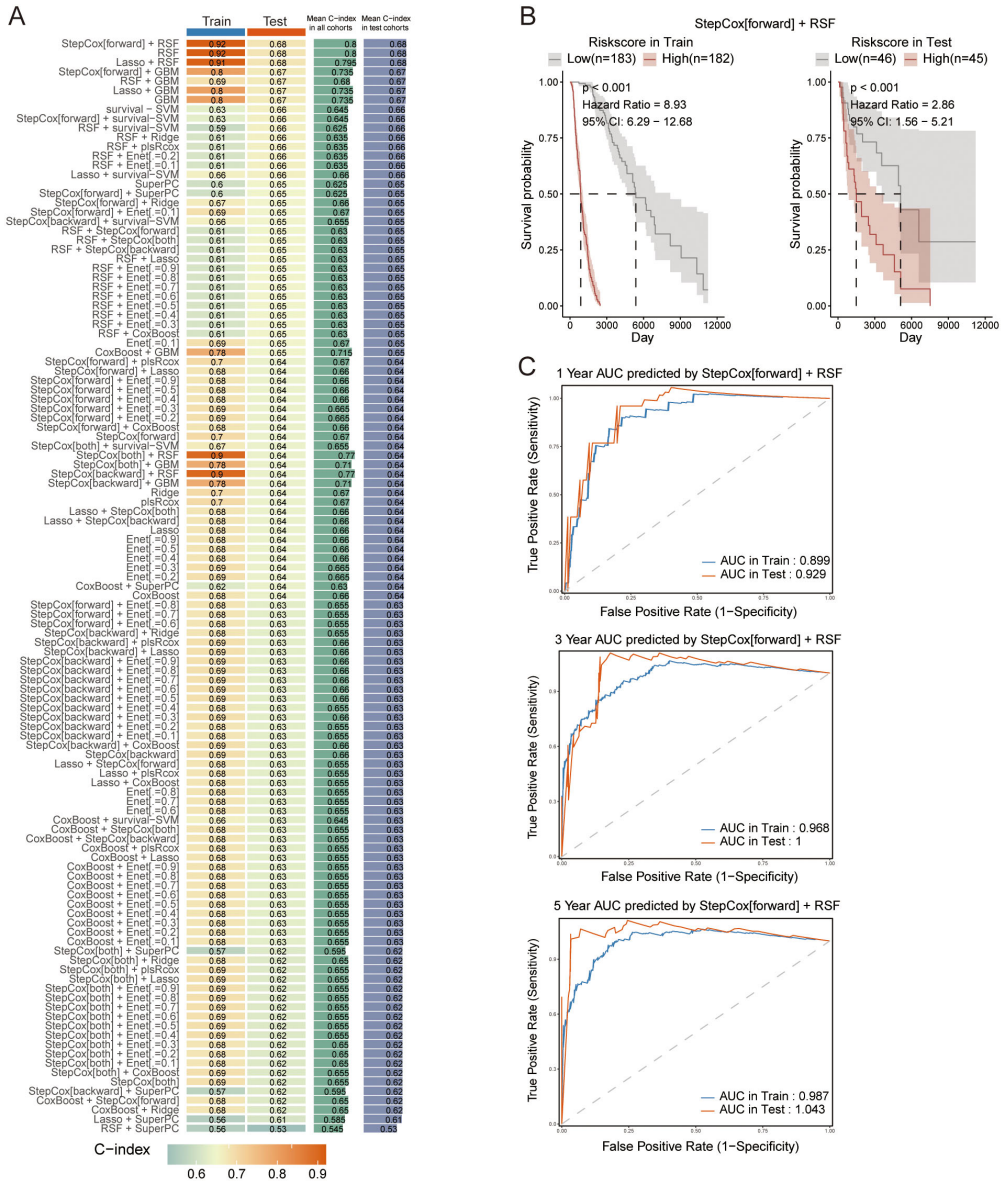
Construction of prognostic models. **(A)** C-index values for each model across both the training and testing datasets, highlighting model performance. **(B)** Survival curves for patients stratified by risk scores, calculated using the StepCox[forward] + RSF combined model across different datasets. **(C)** ROC curves for the 1-, 3-, and 5-year survival predictions, demonstrating the performance of the StepCox[forward] + RSF combined model in both the training and testing datasets.

The TCGA melanoma dataset (n = 456) was divided into a training subset (75%, n = 342) and a testing subset (25%, n = 114). A set of 103 overlapping genes was utilized with both subsets to build prognostic models, applying 10 machine learning algorithms via Mime. Out of the 117 models developed, the StepCox[forward] + RSF combined model achieved the highest C-index mean across both the training and testing datasets (**Figure 3A**). Both the StepCox[forward] + RSF combined model and the RSF model yielded the same mean C-index across training and testing datasets. Since the combined model selected the same feature genes as both methods, which are deemed more significant, we chose the StepCox[forward] + RSF combined model for further analysis. Based on the median risk score calculated by Mime from the combined mode, patients were categorized into high-risk and low-risk groups. The survival probability for each cohort was assessed, showing that individuals in the high-risk group had significantly poorer outcomes in both datasets (**Figure 3B**). Notably, the 3- and 5-year AUC of the combined model reached 1 in the test set and >0.96 in the training set, indicating the model’s exceptional precision and stability (**Figure 3C**).

### 3.3 Selection of significant feature genes

**Figure 4:**
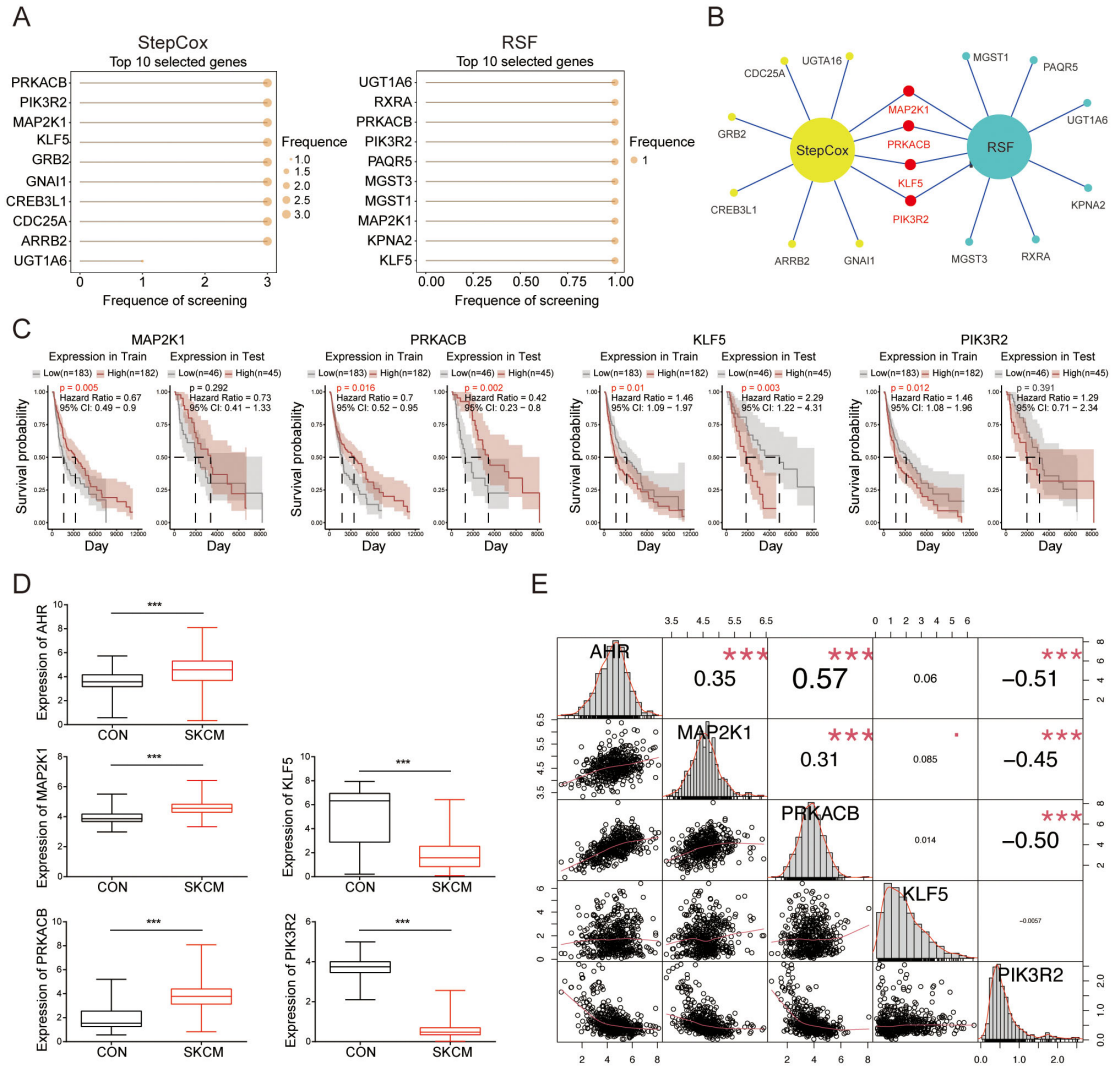
Selection of significant feature genes. **(A)** Top 10 features selected by the StepCox (left) and Random Forest (right) algorithms. **(B)** Venn diagram showing the intersection of four feature genes derived from both the StepCox and Random Forest models. **(C)** Survival curves of patients stratified by the median expression levels of each gene across different datasets. **(D)** Comparison of the expression levels of the identified feature genes in melanoma patients from the TCGA database against normal controls from the GTEx database. **(E)** A correlation heatmap illustrating the relationships among the selected genes. ***P < 0.001.

We analyzed the top 10 genes from the StepCox and RSF models, respectively (**Figure 4A**). Using a Venn diagram, we identified MAP2K1, PRKACB, KLF5, and PIK3R2 as significant features common to both models (**Figure 4B**). Based on the expression of the feature genes, patients were stratified into high-risk and low-risk groups using Mime, and survival probabilities were calculated for each cohort. Notably, higher expression levels of PRKACB and MAP2K1 were associated with better survival outcomes, while elevated expression of KLF5 and PIK3R2 correlated with poorer prognosis (**Figure 4C**). An examination of the expression profiles of melanoma patients from the TCGA database, compared to normal controls from the GTEx database, revealed that AHR, MAP2K1, and PRKACB were upregulated in melanoma, whereas KLF5 and PIK3R2 were downregulated (**Figure 4D**). Correlation analysis demonstrated a significant positive correlation between AHR and MAP2K1, as well as PRKACB, and a significant negative correlation with PIK3R2. No significant correlation was observed between KLF5 and the other genes (**Figure 4E**). AHR also co-expressed with these feature genes in a skin tissue coexpression network (**Supplementary Figure S2**). These results suggest that these genes are not only key prognostic markers but may also play essential roles in melanoma progression, positioning them as promising targets for therapy.

To assess the robustness and predictive accuracy of the prognostic model, we used external datasets, GSE19234 and GSE65904, as independent test sets. The combined StepCox[forward] + RSF model showed strong consistency across these datasets (**Supplementary Figures S3A, C**). Notably, melanoma patients in GSE65904 with high MAP2K1 expression exhibited better outcomes (**Supplementary Figures S3B, D**). Additionally, we conducted a correlation analysis of MAP2K1, KLF5, PRKACB, and PIK3R2 with immune signatures across both datasets (**Supplementary Figures S3E, F**). However, due to the limited sample size, survival differences between the low-risk and high-risk groups were not statistically significant for other genes, and their correlations with immune signatures varied between the datasets.

### 3.4 GSEA analysis of selected feature genes

**Figure 5:**
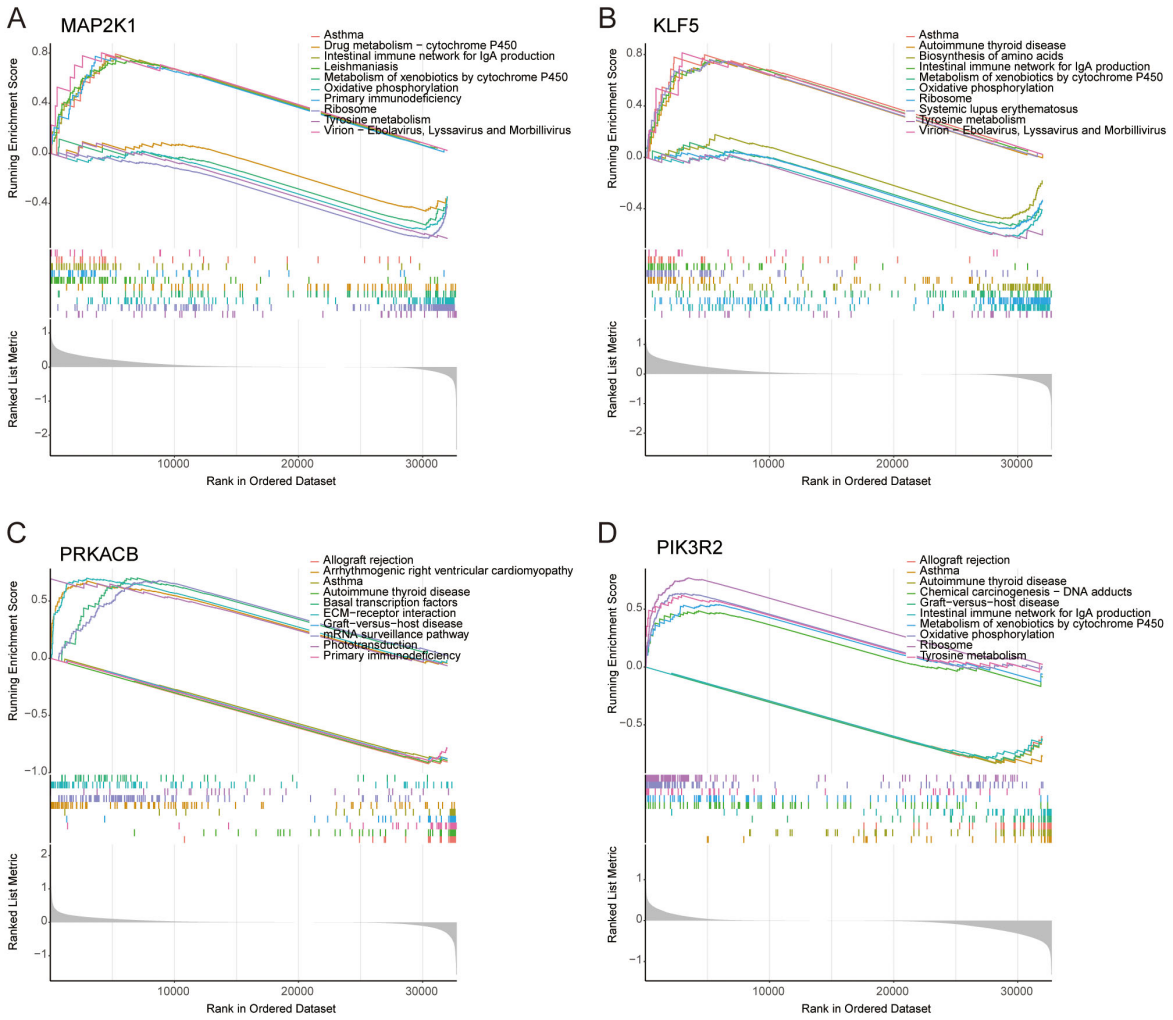
GSEA analysis of selected feature genes. In terms of KEGG pathway analysis, the top five upregulated and downregulated pathways were identified for **(A)** MAP2K1, **(B)** KLF5, **(C)** PRKACB, and **(D)** PIK3R2.

Beyond their prognostic significance, the four feature genes are implicated in key biological pathways. GSEA revealed that MAP2K1 is predominantly associated with the metabolism of xenobiotics by cytochrome P450, primary immunodeficiency and tyrosine metabolism (**Figure 5A**). KLF5 is linked to the metabolism of xenobiotics by cytochrome P450 and tyrosine metabolism (**Figure 5B**). PRKACB is primarily involved in primary immunodeficiency (**Figure 5C**). PIK3R2 plays a role in the metabolism of xenobiotics by cytochrome P450, tyrosine metabolism and oxidative phosphorylation (**Figure 5D**).

### 3.5 Correlation between feature genes and immune signatures

**Figure 6:**
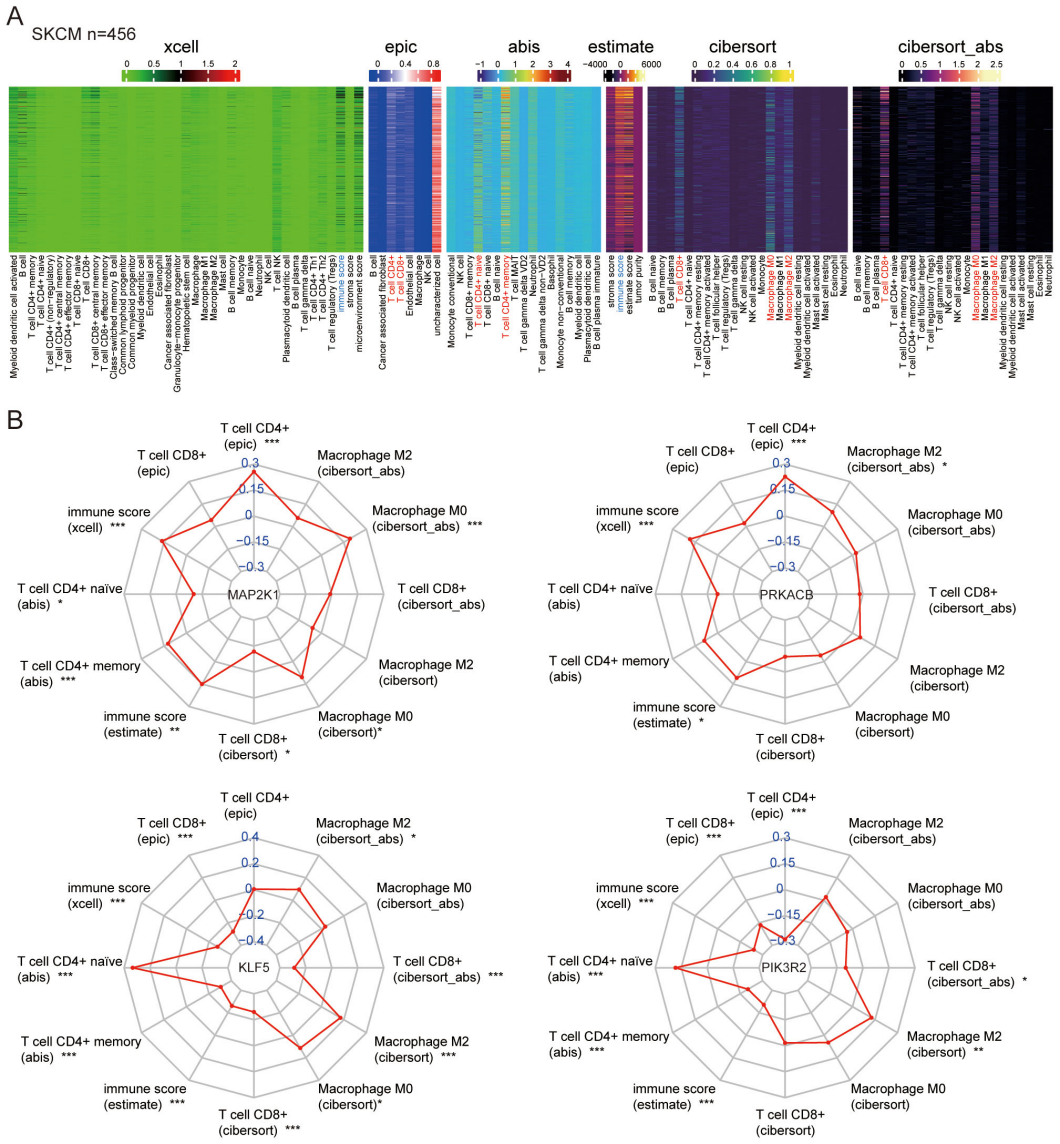
Correlation between feature genes and immune signatures. **(A)** Immune signatures in melanoma samples from TCGA were deconvoluted using various methods. **(B)** Correlations were analyzed between the feature genes (MAP2K1, KLF5, PRKACB, PIK3R2) and the immune signatures. *P < 0.05, **P < 0.01, ***P < 0.001.

The immunological environment plays a critical role in the progression of melanoma. To explore this, we enriched immune infiltration and tumor microenvironment signatures using Mime. Immune scores obtained through various tools, including xCell and ESTIMATE, while specific immune cell populations such as CD4^+^ T cells (via EPIC), CD4^+^ naïve and memory T cells (via ABIS), CD8^+^ T cells, and macrophages (M0 and M2 types) were assessed using CIBERSORT and CIBERSORT_abs, showing high expression in melanoma samples from the TCGA dataset (**Figure 6A**).

Correlation analysis between the feature genes and immune cell signatures revealed that MAP2K1 and PRKACB are positively correlated with CD4^+^ T cells and immune score, both of which are crucial for anti-tumor immunity. KLF5 and PIK3R2 were associated with the CD4^+^ naïve T cells and immunosuppressive macrophages (M2 type) (**Figure 6B**). These findings highlight the diverse roles of AHR-related genes in melanoma and emphasize their potential as key modulators of the immune landscape.

### 3.6 Validation of the relationships between feature genes and immune cells

**Figure 7:**
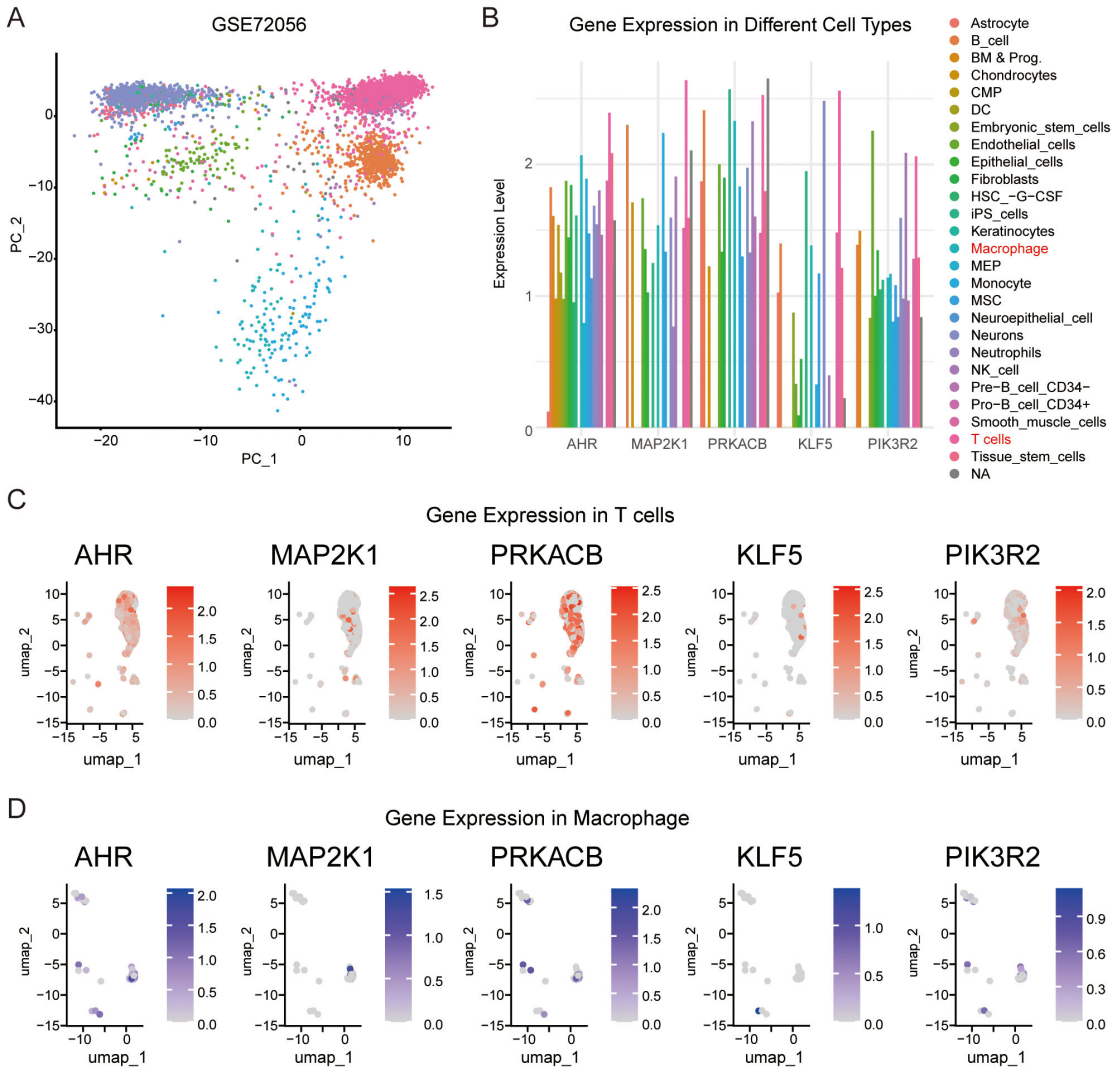
Validation of the relationships between feature genes and immune cells. **(A)** Single cell expression profiles were clustered into different cell types using the SingleR R package. **(B)** Expression levels of AHR and the four feature genes across various cell types. **(C)** Expression of AHR, MAP2K1, KLF5, PRKACB, and PIK3R2 in T cells. **(D)** Expression of AHR, MAP2K1, KLF5, PRKACB, and PIK3R2 in macrophage.

We analyzed the expression of the feature genes in different immune cells using single-cell sequencing data from melanoma (GSE72056). First, we grouped the cells based on their expression profiles (**Figure 7A**) and examined the expression of the feature genes across different cell types (**Figure 7B**). Notably, AHR and the four feature genes were expressed in T cells and macrophages (**Figures 7C, D**). These results further confirm that the feature genes are involved in immune responses within the melanoma microenvironment. The expression of these genes in T cells and macrophages, key immune cell types, supports the hypothesis that they play crucial roles in regulating immune responses and may influence the tumor’s ability to evade immune surveillance.

### 3.7 Validation of the feature genes in mouse melanoma model

**Figure 8:**
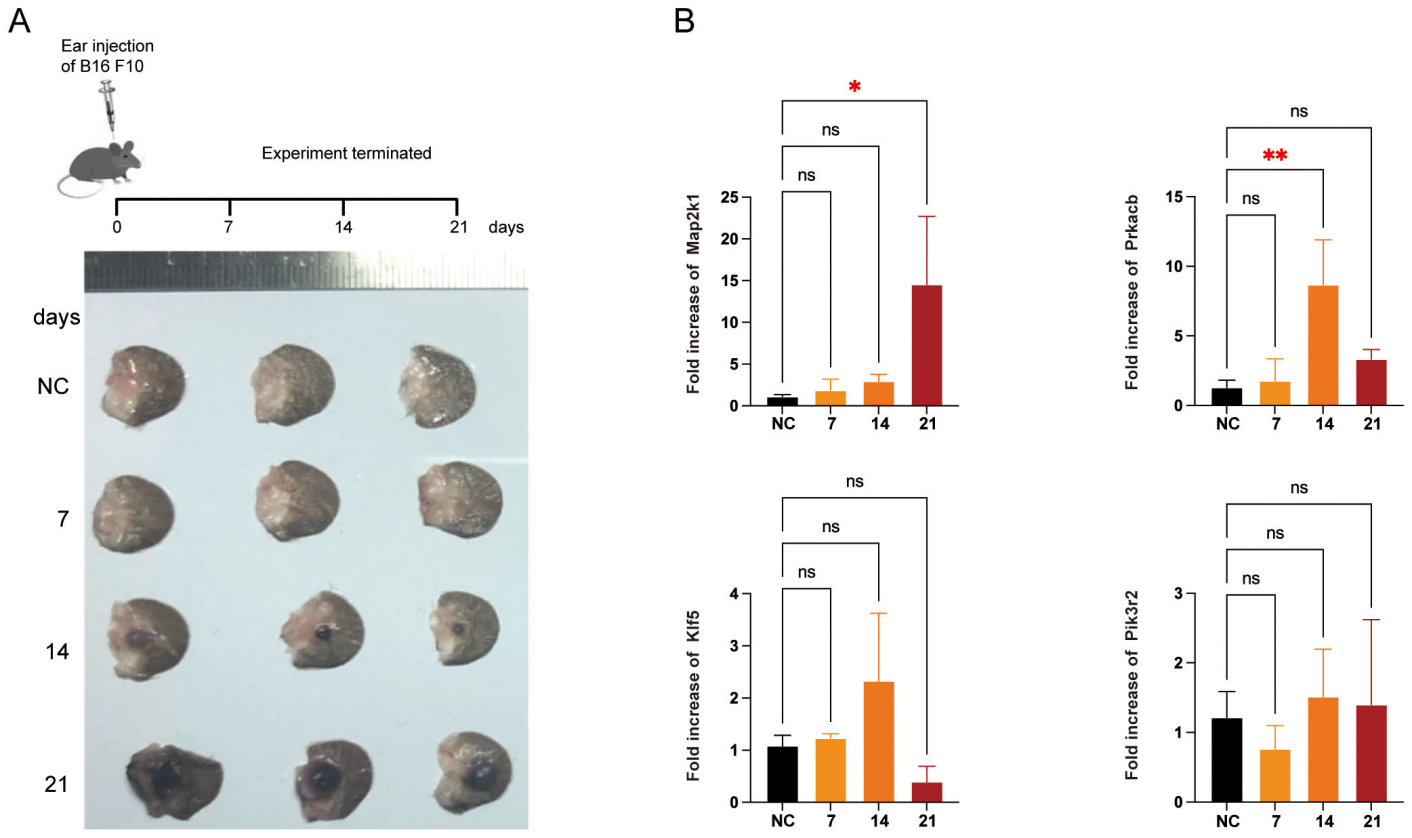
Validation of the feature genes in mice melanoma model. **(A)** Schematic representation for the mice model. B16 F10 cells were injected into right ears of C57BL/6 mice (n = 3). Representative tumor images from three independent experiments were shown. **(B)** qRT-PCR for the feature genes in the tumor or normal tissue. *P < 0.05, **P < 0.01.

To further validate the dynamic changes in the feature genes during melanoma progression, we established a melanoma model in the mouse ear (**Figure 8A**), which is considered more clinically relevant for studying the progression and metastasis of human melanoma (26). In this model, the mRNA levels of Map2k1 and Prkacb increased significantly on days 21 and 14, respectively, whereas the expression of Klf5 and Pik3r2 showed no significant changes (**Figures 8B**).

### 3.8 Construction of mRNA-miRNAs-lncRNAs network and Drug-gene interactions

We predicted the drugs correlated with AHR, MAP2K1, KLF5, PRKACB and PIK3R2 using data from the CTRP and GDSC databases. Additionally, the drug-gene interactions were visualized using the GSCA platform (**Supplementary Figures S4A, B**). To further explore regulatory mechanisms, we searched the miRDB database to identify miRNAs with a target score of ≥ 95 that are linked to the mRNAs of these feature genes. We then used the ENCORI database to identify lncRNAs associated with these miRNAs. A comprehensive mRNA-miRNA-lncRNA network was constructed by intersecting the identified miRNAs and lncRNAs (**Supplementary Figure S5**). These analyses aim to uncover potential therapeutic strategies for melanoma by targeting key molecules in the AHR, MAP2K1, KLF5, PRKACB, and PIK3R2 signaling pathways. These analyses aim to identify therapeutic strategies for melanoma by targeting key genes (AHR, MAP2K1, KLF5, PRKACB, PIK3R2) and their regulatory RNA networks, offering potential for personalized treatments and novel biomarkers to improve clinical outcomes.

## 4 Discussion

### 4.1 Overview of findings

This study provides significant insights into the role of the AhR pathway in melanoma, particularly in relation to tumor progression and immune modulation. Previous research has highlighted the dualistic nature of AhR signaling in cancer, where it can either suppress or promote tumorigenesis depending on the context (5, 27). Recent evidences in melanoma, where AhR appears to promote macrophage polarization towards immunosuppressive phenotype (11) and widely suppress immune cell function (28), complicating its utility as a straightforward therapeutic target. By integrating bioinformatics and machine learning, we identified four key AhR-associated genes—MAP2K1, PRKACB, KLF5, and PIK3R2—that serve as both prognostic markers and potential therapeutic targets. These genes were systematically analyzed for their biological roles and correlations with immune infiltration and patient outcomes.

### 4.2 Biological and clinical implications of key genes

MAP2K1, also referred to as MEK1, is a crucial element of the mitogen-activated protein kinase (MAPK) signaling pathway, which plays a pivotal role in controlling cell proliferation, differentiation, and survival (29, 30). MEK1/2 can activated AhR, resulting in a transient inhibition of cytochrome P450 family 1 subfamily A member 1 (CYP1A1) (31). AHR introduces the transcriptional activation of CYP1A1, which facilitates the biotransformation of environmental toxins and carcinogenic substances into highly reactive and carcinogenic diol epoxide intermediates (32). Additionally, AhR can influence MAPK signaling, thereby affecting cellular processes such as dysfunction and apoptosis (33). In melanoma, dysregulation of MAPK signaling, often through mutations in upstream effectors like BRAF, leads to unchecked tumor growth and resistance to therapy (34). Our results demonstrate a positive association between MAP2K1 expression and T cell infiltration in the TME and better outcomes. This finding is significant because it underscores the complex role of MAP2K1, which could potentially be exploited to enhance the efficacy of immunotherapies.

PRKACB, a gene encoding the catalytic subunit of protein kinase A (PKA), is involved in the regulation of metabolism, transcription, and immune response (35). Studies show that AhR can activate PKA signaling, thereby regulating the activation of cancer stemness (36). In melanoma, PKA can enhance the migration and metastasis of melanoma cells (37) and impair T cell infiltration into the tumor microenvironment (38). Recent evidence indicated that PKA mediates the growth inhibition of melanoma cells (39). In our study, the positive correlation between PRKACB expression and immune score, suggests that PRKACB may facilitate anti-tumor immunity in melanoma. PKA has been shown to modulate can phosphorylate the NF-κB subunit p65, promoting T cell activation and survival (40, 41), which may explain the association between high PRKACB expression and better survival outcomes observed in this study. Interestingly, PRKACB is also associated with inhibiting the proliferation and invasion of tumor cells (42), making it a promising therapeutic target in combination with existing immunotherapies.

KLF5 (Kruppel-like factor 5) is a transcription factor known for its role in cell proliferation, differentiation, and apoptosis (43). Studies indicate that KLF5 can enhance the expression of CYP1A1, which are involved in inducing the expression of proinflammatory cytokines (such as TNF) that can influence melanoma progression (44, 45). KLF5 can promote the epithelial-mesenchymal transition (EMT) (46), a process crucial for melanoma invasion and metastasis (47), making it a potential target for therapeutic interventions aimed at disrupting these pathways. Moreover, KLF5 can promote the podosome formation in macrophages and enhance the tissue infiltration ability of macrophages (48). KLF5 promotes malignant phenotype of melanoma cells and inhibits autophagy, leading to poor prognosis (49). Targeting KLF5 could, therefore, be a potential strategy to reverse EMT and reduce immunosuppression in the TME.

PIK3R2, encoding the regulatory subunit of phosphoinositide-3-kinase (PI3K), is frequently activated in various cancers, including melanoma (50). Activation of AHR has been shown to lead to the phosphorylation of AKT, a downstream effector of the PI3K pathway, thereby promoting tumor cell proliferation and chemotherapy drug resistance (32). PIK3R2 is identified to promote malignant progression of melanoma by activating the PI3K/AKT/NF - κ B pathway (51). Here, we indicate a negative correlation between PIK3R2 expression and T cell infiltration, alongside a positive association with M2 macrophages. This dual association highlights the immunosuppressive role of PIK3R2 in the TME. Additionally, several bioinformatics analyses based on melanoma transcriptome have indicated that PIK3R2 leads to poor prognosis and low immune cell infiltration in melanoma (52, 53). Targeting PIK3R2 in combination with therapies that reprogram macrophages could potentially enhance anti-tumor immunity and improve patient outcomes.

The upregulation of AHR, MAP2K1, and PRKACB, alongside the downregulation of KLF5 and PIK3R2 (**Figure 4D**), suggests that these genes play critical roles in melanoma progression through various signaling pathways. AHR promotes immune evasion and tumor progression, influencing MAP2K1 and PRKACB, which regulate key pathways like MAPK and NF-κB signaling, respectively. Conversely, the downregulation of KLF5 and PIK3R2 may facilitate a more aggressive, proliferative melanoma phenotype by affecting cell differentiation and metabolism (**Figure 5**). In the mouse model, the differential expression of these genes over time reflects their roles in tumor progression, with the immune microenvironment possibly influencing gene expression. These findings highlight the importance of these genes as potential therapeutic targets and the need for further investigation into their roles in immune modulation and melanoma progression.

### 4.3 Integration of bioinformatics and machine learning

The extensive application of high-throughput sequencing technologies and machine learning has significantly advanced our comprehension of biological processes and cancer heterogeneity (54). Increasingly, researchers have been able to identify distinct molecular characteristics associated with disease progression, patient outcomes, and responses to treatment using sequencing data (55). By leveraging diverse feature selection algorithms, the study achieved high C-index values, validating the reliability of these genes in predicting patient survival outcomes. This integration underscores the growing potential of computational tools in uncovering complex molecular interactions and identifying actionable therapeutic targets. Additionally, Drug sensitivity analyses further support the feasibility of these approaches, providing a foundation for preclinical and clinical investigations.

### 4.4 Limitations and future directions

However, our study has certain limitations. A more profound understanding of the molecular mechanisms linking AHR with melanoma is evidently required. Both *in vivo* and *in vitro* experiments hold great potential to clarify these complexities, indicating numerous opportunities for future research. This inquiry is anticipated to expand our knowledge and introduce new therapeutic possibilities for managing melanoma, paving the way for enhanced understanding and future innovations in treatment.

### 4.5 Conclusion

This study provides an integrated approach to understanding the AhR pathway’s role in melanoma, identifying MAP2K1, PRKACB, KLF5, and PIK3R2 as critical prognostic markers and therapeutic targets. By employing bioinformatic tools and machine learning techniques, a more detailed understanding of the AHR pathway’s involvement in immune regulation and tumor development has been achieved. The use of bioinformatics and machine learning not only enhances our understanding of melanoma biology but also paves the way for more effective therapeutic strategies.

## Data Availability

The original contributions presented in the study are included in the article/**Supplementary Material**. Further inquiries can be directed to the corresponding author.
