# Supplementary material for: Integrating bioinformatics and machine learning to identify AhR-related gene signatures for prognosis and tumor microenvironment modulation in melanoma
Source: Front Immunol. 2025 Jan 6;15:1519345. doi: 10.3389/fimmu.2024.1519345 (PMC11743449; doi:10.3389/fimmu.2024.1519345)
Supplement: Supplementary file 1 [file DataSheet1.docx]

**Figure S1**


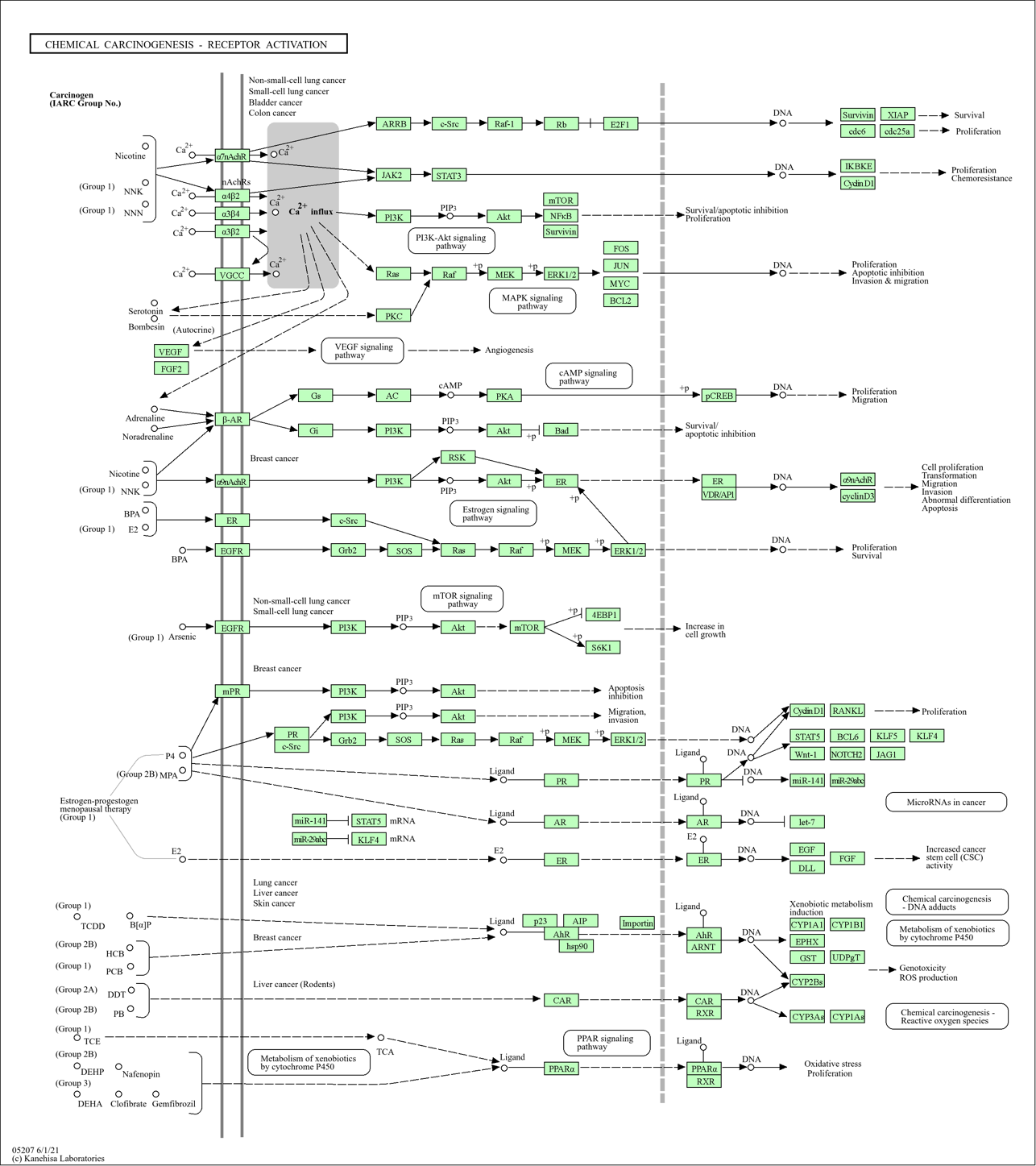


**Figure S1** Chemical carcinogenesis - receptor activation (hsa05207). The channel information and images are sourced from https://www.kegg.jp/entry/hsa05207.

**Figure S2**


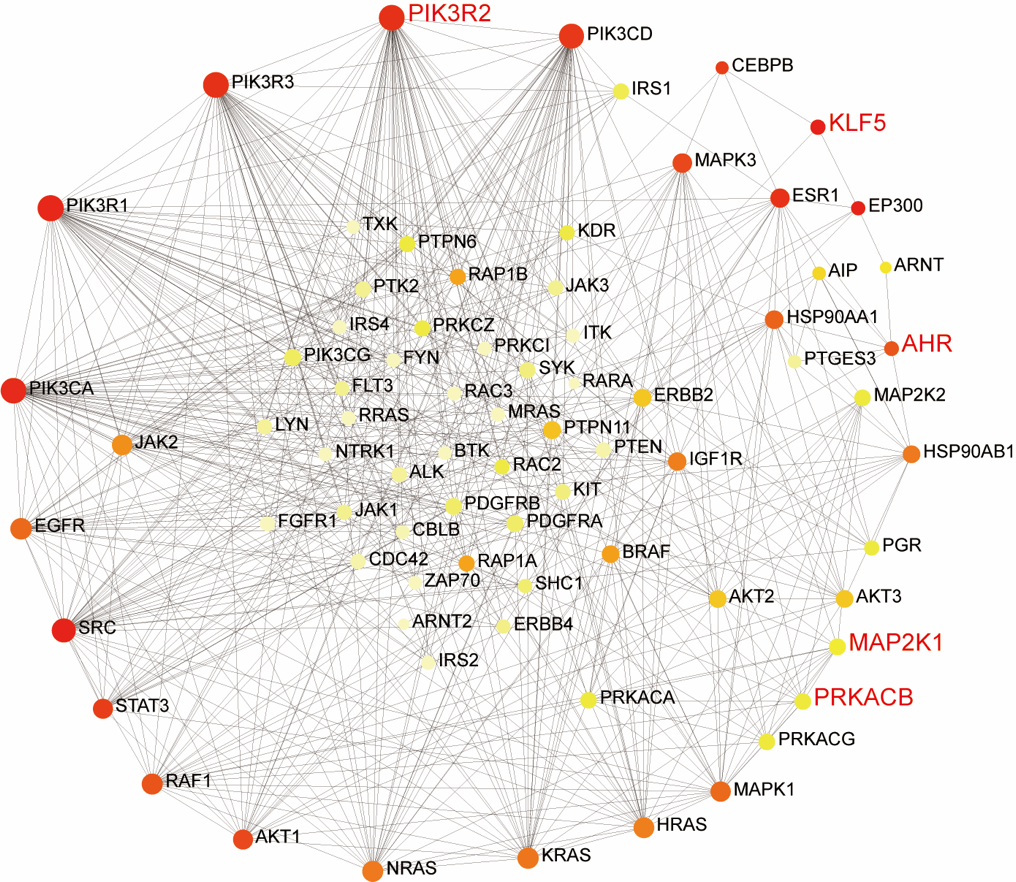


**Figure S2** The skin-specific coexpression network of the 103 genes. Zero-order interaction network of the genes in skin. The colors of nodes are positively correlated with the number of node-neighbors.

**Figure S3**

**
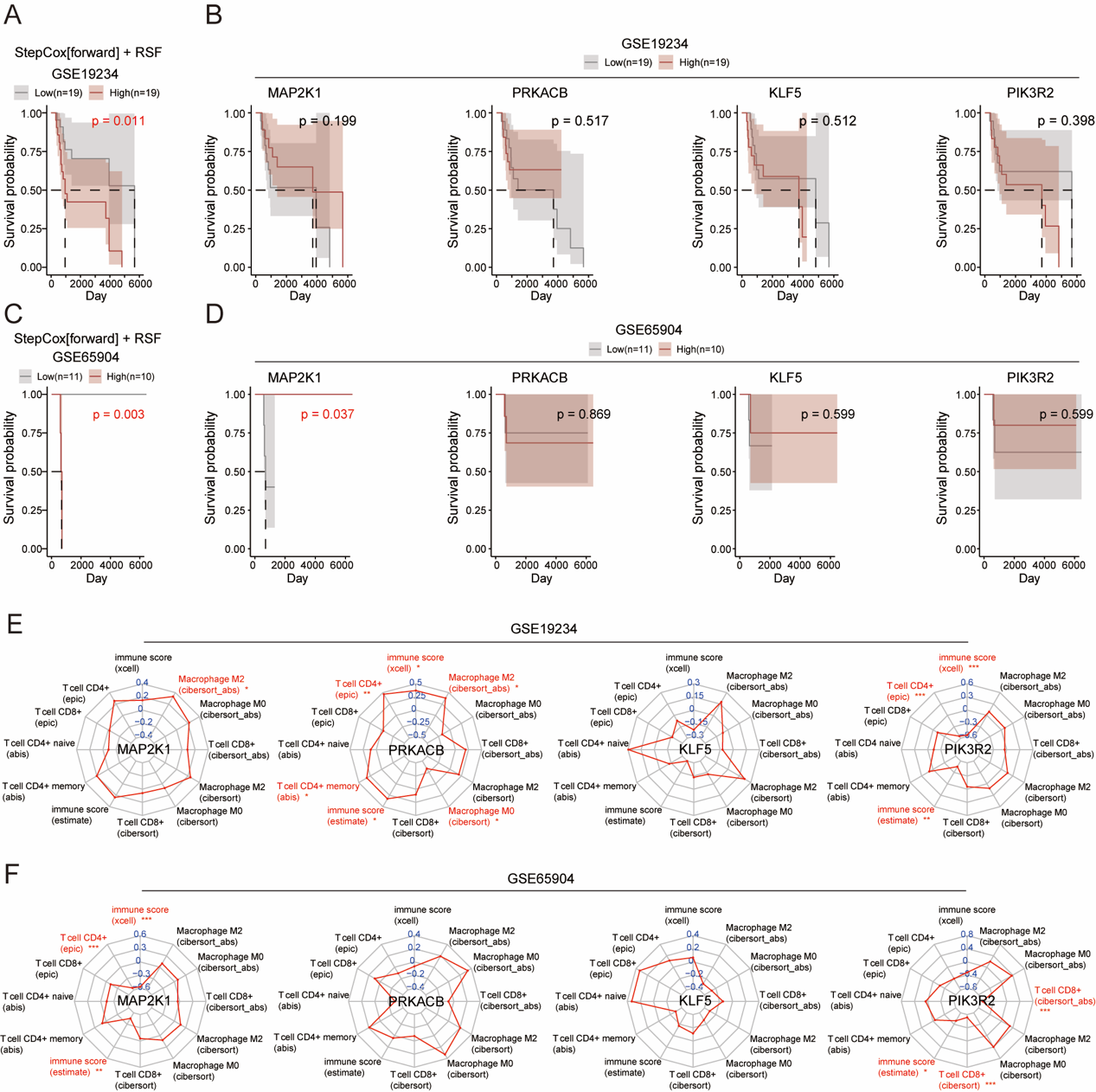
**

**Figure S3** Validation of the model and four feature genes. (A) StepCox[forward] + RSF combined mode confidence and (B) prediction accuracy of four features of GSE19234. (C) StepCox[forward] + RSF combined mode confidence and (D) prediction accuracy of four features of GSE65904. Correlations between MAP2K1, KLF5, PRKACB, PIK3R2 and immune signatures of GSE19234 (E) and GSE65904 (F).

**Figure S4**


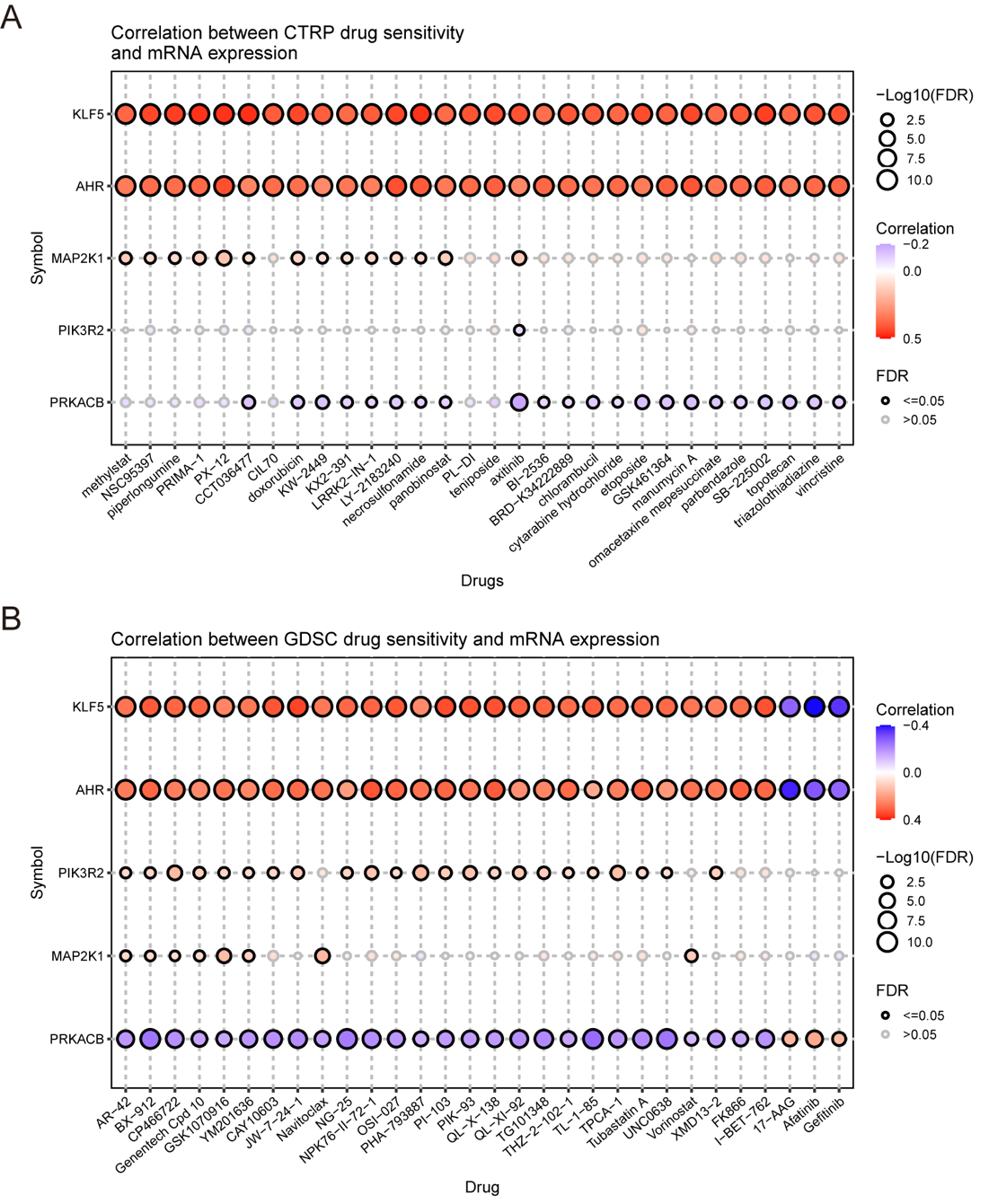


**Figure S4** Drug-gene interactions. AHR, MAP2K1, KLF5, PRKACB and PIK3R2 associated drugs from (A) CTRP and (B) GDSC database.

**Figure S5**


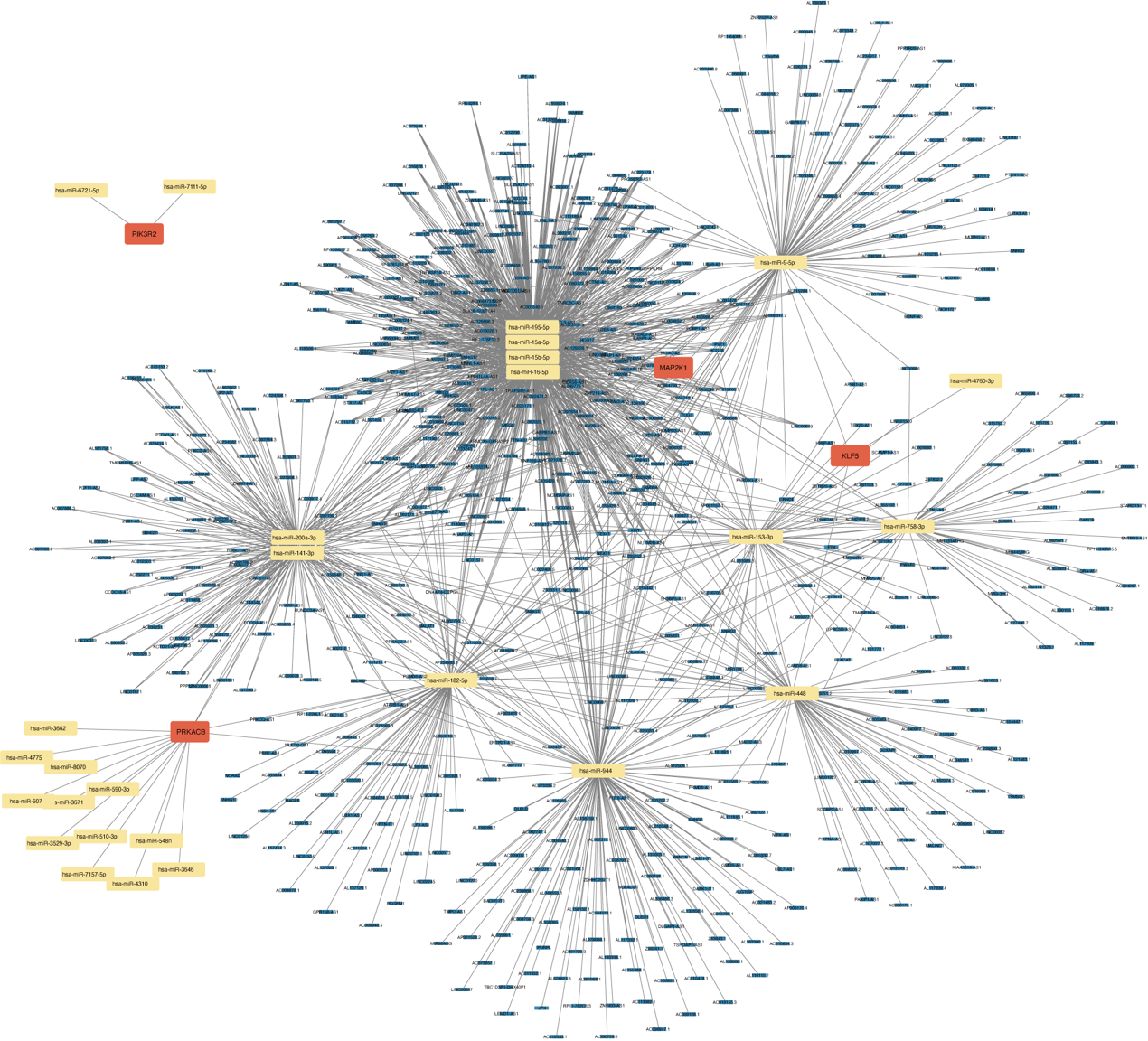


**Figure S5** Construction of mRNA-miRNAs-lncRNAs network. Red boxes are mRNAs, yellow boxes are miRNAs, and blue boxes are lncRNAs.
